# Comparison of Endoscopic Ultrasonography and Multislice Spiral Computed Tomography for the Preoperative Staging of Gastric Cancer - Results of a Single Institution Study of 610 Chinese Patients

**DOI:** 10.1371/journal.pone.0078846

**Published:** 2013-11-01

**Authors:** Xing-yu Feng, Wei Wang, Guang-yu Luo, Jing Wu, Zhi-wei Zhou, Wei Li, Xiao-wei Sun, Yuan-fang Li, Da-zhi Xu, Yuan-xiang Guan, Shi Chen, You-qing Zhan, Xiao-shi Zhang, Guo-liang Xu, Rong Zhang, Ying-bo Chen

**Affiliations:** 1 State Key Laboratory of Oncology in South China, Guangzhou, China; 2 Department of Gastric and Pancreatic Surgery, Sun Yat-sen University Cancer Center, Guangzhou, China; 3 Department of Endoscopy and Laser, Sun Yat-sen University Cancer Center, Guangzhou, China; 4 Department of Interventional Radiology and Medical Imaging, Sun Yat-sen University Cancer Center, Guangzhou, China; 5 Department of Melanoma Medical Oncology, Sun Yat-sen University Cancer Center, Guangzhou, China;; Virginia Commonwealth University School of Medicine, United States of America

## Abstract

**Background:**

This study compared the performance of endoscopic ultrasonography (EUS) and multislice spiral computed tomography (MSCT) in the preoperative staging of gastric cancer.

**Methodology/Principal Findings:**

A total of 610 patients participated in this study, all of whom had undergone surgical resection, had confirmed gastric cancer and were evaluated with EUS and MSCT. Tumor staging was evaluated using the Tumor-Node-Metastasis (TNM) staging and Japanese classification. The results from the imaging modalities were compared with the postoperative histopathological outcomes. The overall accuracies of EUS and MSCT for the T staging category were 76.7% and 78.2% (P=0.537), respectively. Stratified analysis revealed that the accuracy of EUS for T1 and T2 staging was significantly higher than that of MSCT (P<0.001 for both) and that the accuracy of MSCT in T3 and T4 staging was significantly higher than that of EUS (P<0.001 and 0.037, respectively). The overall accuracy of MSCT was 67.2% when using the 13th edition Japanese classification, and this percentage was significantly higher than the accuracy of EUS (49.3%) and MSCT (44.6%) when using the 6th edition UICC classification (P<0.001 for both values).

**Conclusions/Significance:**

Our results demonstrated that the overall accuracies of EUS and MSCT for preoperative staging were not significantly different. We suggest that a combination of EUS and MSCT is required for preoperative evaluation of TNM staging.

## Introduction

Gastric cancer is one of the most common malignant tumors in the digestive tract and is the second most common cause of cancer-related death worldwide [1]. Despite a decline in morbidity and mortality, gastric cancer still occurs frequently and poses a severe threat to human health, particularly in China. The primary management of gastric cancer is surgical resection [2], but new therapeutic approaches have been developed in the past two decades. Endoscopic mucosal resection (EMR) or endoscopic submucosal dissection (ESD) can be performed in patients with early gastric cancer (EGC) to avoid further unnecessary invasive surgical procedures [3]. Neoadjuvant treatments have been investigated to achieve R0 resection for local advanced gastric cancer (AGC) [4]. Therefore, precision in preoperative staging is essential to the individualized stage-dependent treatment of gastric cancer and to the prognosis of patients with gastric cancer.

Endoscopic ultrasonography (EUS) and multislice spiral computed tomography (MSCT) are the most common techniques for the preoperative staging of gastric cancer patients. The predictive value of these two techniques has been the focus of research since the 1990s [5–13]. However, the results of past studies are conflicting. While some studies have reported that EUS is superior to MSCT [5,6], other studies have indicated that the accuracy of MSCT has greatly improved and that its predictive value is similar to that of EUS [9,12]. Furthermore, some of these reports focused on EGC, while others focused on AGC.

The progression of clinical techniques and improvements in clinician experience necessitate new reports in this field. A large-sample direct comparison study of EUS and MSCT for T, N, and M staging in patients with EGC and AGC should be performed to build upon previous evidence with relatively small sample sizes and conflicting results. We hypothesize that EUS and MSCT differ with regards to preoperative accuracy and possess different advantages in staging. Therefore, both modalities were compared in a relatively large sample population at a center in China to evaluate which one is more reliable in different staging patients and to provide solid evidence for treatment strategies. 

## Methods

### Ethics statement

The protocol was approved by the Sun Yat-sen University Cancer Center review board, in accordance with Chinese bioethical regulations. All patients provided written informed consent before participating in the study.

### Participants

A total of 610 patients who had undergone surgical resection at the Sun Yat-Sen University Cancer Center from October 2006 to March 2011 and who had confirmed gastric cancer participated in this study. Patients who had received EMR or ESD and patients with widespread metastatic disease who had not yet undergone surgical resection were excluded from the study. Given the potential influence of the accuracy of MSCT and EUS caused by the changes in the primary tumor and the lymph nodes , patients who had received pre-operative therapy were also excluded. All patients received preoperative TNM staging using EUS and MSCT during the two weeks prior to surgery. The imaging, clinical and postoperative pathological materials of the patients were available. 

### EUS

**Figure 1:**
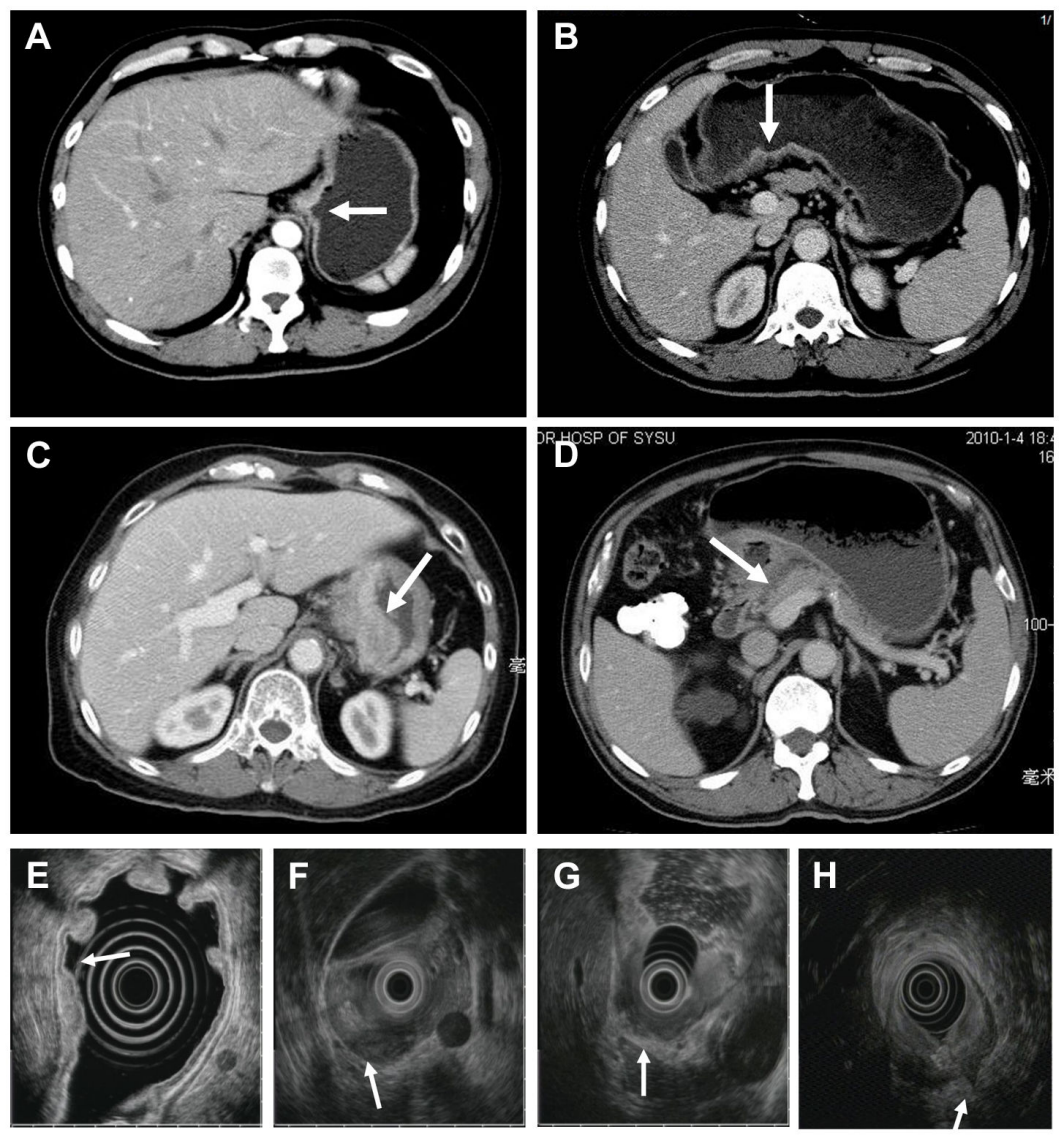
T staging using MSCT and EUS. **A**. MSCT-T1 tumor: Transverse CT image shows an elevated lesion (arrow) of the gastric mucosa of the lesser curvature with a clear fat plane. The elevated gastric mucosa shows strong enhancement, and the tumor is confined to the mucosa. **B**. MSCT-T2 tumor: Transverse CT image shows an elevated lesion (arrow) of the gastric mucosa of the lesser curvature with a clear fat plane. The elevated gastric mucosa shows strong enhancement, and the tumor is considered as the invasion into the muscular layer but not the serosa. **C**. MSCT-T3 tumor: Transverse CT image shows a markedly thickened gastric wall (arrow) of the lesser curvature. The tumor extends beyond the serosal layer and affects the fat plane. Its relation with adjacent organs can be distinguished. **D**. MSCT-T4 tumor: Transverse CT image of a transmural tumor of the gastric antrum (arrow) with a markedly thickened gastric wall and invasion of the head of the pancreas. **E**. EUS-T1 cancer: Endosonographic image of T1 gastric cancer showing hypoechogenic wall thickening with infiltration of the mucosal and submucosal layers (arrow). **F**. EUS-T2 cancer: Gastric carcinoma with infiltration of the muscularis propria (arrow). **G**. EUS-T3 cancer: Transmural hypoechoic tumor with penetration into serosa (arrow). **H**. EUS-T4 cancer: EUS showing advanced gastric cancer with infiltration of the head of the pancreas (arrow).

**Figure 2:**
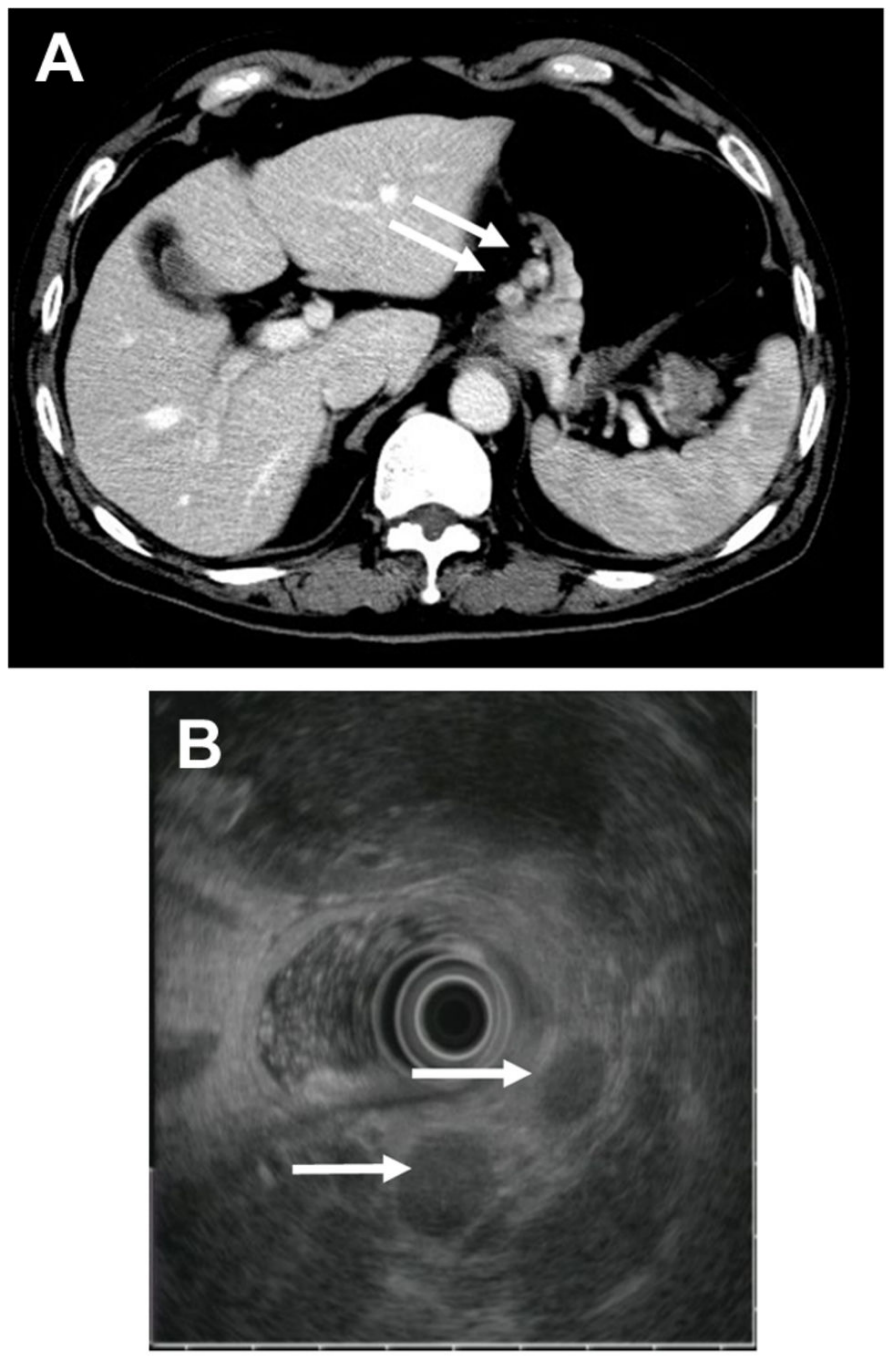
N staging using MSCT and EUS. **A**. MSCT- Lymph node metastases: Transverse CT image of two enlarged lymph nodes (arrows) near the left gastric artery, which shows strong enhancement on enhanced scanning and areas of necrosis in the nodes. **B**. EUS- Lymph node metastases: EUS showing two hypoechoic lymph nodes (arrows), the largest of which is 16.2×14.3 mm.

An AUM-2000 set at variable frequencies of 5, 7.5, 15, and 20 MHz and a UM-2R miniprobe set at 12 or 20 MHz (Olympus, Tokyo, Japan) were used for the EUS examinations. Conventional gastroscopic inspection equipment, including a GIF-XQ240 and a GIF-XQ260 (Olympus, Tokyo, Japan), was used. Patient preparation for EUS was identical to that for conventional endoscopy. Conventional endoscopy was performed to obtain general information about the stomach, and clean food residues and mucus. Endoscopic ultrasonography was performed proximal to the descending portion of the duodenum, except in patients with gastric outlet obstruction. We inspected the stomach during echoendoscope removal. De-aerated water was instilled to improve the transmission of the ultrasound beam. Acoustic coupling with the gastric wall was obtained by instilling 500-800 ml of de-aerated water into the gastric cavity or 200-500 ml into the duodenum. The ultrasonic aspect of tumors and their contiguous structures were assessed by moving the endoscope tip along the entire stomach. The pancreatic body and tail, spleen, splenic hilar lymph nodes, left liver and hilus hepatis lymph nodes, stomach retinal left and right lymph nodes, stomach left and right lymph nodes, celiac lymph nodes, cardiac lymphatic loop and subcarinal lymph nodes were successively assessed. Generally, endoscopic ultrasonography was used to scan larger lesions and lymph nodes. The miniprobe was used with endoscopic ultrasonography for relatively smaller lesions. The same group of expert endoscopists performed endoscopic ultrasonography imaging. Local tumor infiltration was determined using the five-layer structure of the gastric wall [14,15]. Briefly, the mucosal (M) layer was visualized as a combination of the first and second hypoechoic layers, and the submucosal (SM) layer corresponded to the third hyperechoic layer. The muscularis propria (MP) layer was visualized as the fourth hypoechoic layer, while the fifth hyperechoic layer included the serosa and subserosa (Figure **1** **. E, F, G, H**). EUS assessment of the N stage was based on the number of metastatic perigastric lymph nodes. A lymph node metastasis was established using two or more of the following criteria: (1) size greater than 5 mm, (2) round shape, (3) hypoechoic pattern, and (4) smooth border [16,17] (Figure **2** **. B**). T and N staging were assessed using the 6th UICC classification [18].

### MSCT

CT examinations were performed using a 16-slice spiral CT (Brilliance TM16, Philips Medical Systems, the Netherland) or a 64-slice spiral CT (Aquilion TSX-101A, Toshiba Medical System, Tokyo, Japan). In the entire cohort comprising 610 patients, only 48 cases underwent imaging using the 16-slice scanners. Based on radiological expert opinion, the use of a 16-slice scanner or a 64-slice scanner does not affect pre-operative staging of gastric cancer. The imaging process was performed according to a standard imaging protocol. Briefly, patients consumed a liquid diet on the day prior to examination. All patients received 600-800 ml water orally 30 minutes prior to imaging. An unenhanced scan was obtained at 120 kV, 250 mA. The scanning layer thickness was 5 mm with a 1-mm pitch, and the scan area included the diaphragmatic domes to the inferior pole of the kidney. Intravenous nonionic contrast material (1.5 ml iopromide per kilogram of body weight, Ultravist 370; Schering, Berlin, Germany) was administered into the antecubital vein at 2.5 ml/s via a high-pressure syringe. Dual-phasic helical scans were obtained at 25 seconds (arterial phase) and 50 seconds (portal-venous phase). The depth of tumor infiltration into the gastric wall (T category) on CT images was classified according to previously described criteria [19–21] (Figure **1** **. A, B, C, D**). Lymph nodes were considered positive for metastasis when the short-axis diameter was larger than 6 mm for perigastric lymph nodes and larger than 8 mm for the extraperigastric lymph nodes, especially nodes with a rounded shape and enhancement on contrast-enhanced CT that were sometimes necrotic (Figure **2** **. A**). N staging was assessed using the 6th edition UICC classification [18] and the 13th edition Japanese classification [22]. Furthermore, all gastroenterologists and radiologists were blinded to the findings of the other staging modality.

### Analysis of results

The results of preoperative staging using EUS and MSCT were compared to the postoperative histological diagnosis. The chi-square test or Fisher’s exact test was used. A *P*-value less than 0.05 was considered statistically significant. All calculations were performed using SPSS 16.0 for Windows software (SPSS, Chicago).

## Results

The study group included 610 patients: 482 (79.1%) males and 128 (20.9%) females. The median age of the patients was 57 years (range: 22-84 years). The group included 48 patients with early gastric cancer (EGC) and 562 patients with advanced gastric cancer (AGC). A total of 272 (44.6%) tumors were located in the upper third of the stomach, 93 (15.3%) tumors were localized in the middle third, 232 (38.0%) tumors were located in the lower third, and 13 (2.1%) tumors were located in the whole stomach. The clinical and pathological characteristics of the enrolled patients are summarized in Table 1. 

**Table 1:** Clinical and pathological characteristics of enrolled subjects.

**Clinical and pathological characteristics**	**Variable**
**Age (years)**	
Range	22-84
Median	57
**Gender**	
Male	482 (79.1%)
Female	128 (20.9%)
**Tumor location**	
Upper stomach	272 (44.6%)
Middle stomach	93 (15.3%)
Lower stomach	232 (38.0%)
Whole stomach	13 (2.1%)
**Property of operation**	
Radical resection	523 (85.7%)
Palliative resection	87 (14.3%)
**Borrmann classification**	
I	28 (5.0%)
II	292 (52.0%)
III	211 (37.5%)
IV	31 (5.5%)
**Pathologic grade**	
Highly differentiated	16 (2.6%)
Middle differentiated	145 (23.8%)
Poorly differentiated	380 (62.3%)
Undifferentiated	69 (11.3%)
**Depth of tumor invasion**	
T1	48 (7.9%)
T2	66 (10.8%)
T3	466 (76.4%)
T4	30 (4.9%)
**Lymph node metastasis**	
N0	185 (30.3%)
N1	198 (32.5%)
N2	144 (23.6%)
N3	83 (13.6%)
**Distant metastasis**	
M0	544 (89.2%)
M1	66 (10.8%)

### Staging accuracy of tumor infiltration (T category) by EUS and MSCT

The results of preoperative T staging using EUS and MSCT are listed in Table 2. The overall accuracies of T staging using EUS and MSCT were 76.7% and 78.2%, respectively, when compared to the postoperative histopathological outcomes. No significant difference between the two techniques was observed (P=0.537). However, these techniques exhibited different advantages for different categories of T staging. The diagnostic accuracy of EUS for T1 and T2 staging was significantly higher than that of MSCT (T1, 83.3% vs. 20.8%; T2, 80.3% vs. 36.4%; P<0.001 for both). Conversely, the accuracy of EUS for T3 and T4 staging was significantly lower than that of MSCT (T3, 76.4% vs. 89.5%; T4, 63.3% vs. 86.7%; P<0.001 and P=0.037, respectively).

**Table 2:** Comparison of preoperative T staging accuracy between EUS and MSCT.

**Pathological stage**	***N***	**EUS**				**Accuracy %**	**MSCT**				**Accuracy %**	***P* value**
		**T1**	**T2**	**T3**	**T4**		**T1**	**T2**	**T3**	**T4**		
**T1**	48	40	7	1	0	83.3	10	31	7	0	20.8	<0.001
**T2**	66	4	53	9	0	80.3	0	24	42	0	36.4	<0.001
**T3**	466	0	86	356	24	76.4	0	40	417	9	89.5	<0.001
**T4**	30	0	3	8	19	63.3	0	0	4	26	86.7	0.037
**Total**	610	44	149	374	43	76.7	10	95	470	35	78.2	0.537

### Staging accuracy of lymph node involvement (N category) using EUS and MSCT

N staging using the 6th edition UICC TNM staging system [18] was accurate in 49.3% (301/610) of EUS studies and 44.6% (272/610) of MSCT studies. No significant difference between the two techniques was observed (P=0.096). Accuracy was decreased for higher N-stages. The accuracy of EUS for preoperative N0, N1, N2 and N3 staging was 75.7%, 58.6%, 27.8% and 6.0%, respectively. The accuracy of MSCT for preoperative N0, N1, N2 and N3 staging was 61.1%, 48.5%, 38.9% and 8.4%, respectively. EUS demonstrated greater accuracy than MSCT for N0 and N1 staging (N0, 75.7% vs. 61.1%; N1, 58.6% vs. 48.5%; P=0.003 and 0.044). In contrast, EUS exhibited lower accuracy for N2 staging than MSCT (27.8% vs. 38.9%, P=0.046). However, the accuracy of EUS for N3 staging was similar to that of MSCT (6.0% vs. 8.4%, P=0.549). The results of preoperative N staging using EUS and MSCT are presented in Table 3. 

**Table 3:** Comparison of preoperative N staging accuracy between EUS and MSCT according to the 6th edition of UICC classification.

**Pathological stage**	***N***	**EUS**				**Accuracy %**	**MSCT**				**Accuracy %**	***P* value**
		**N0**	**N1**	**N2**	**N3**		**N0**	**N1**	**N2**	**N3**		
**N0**	185	140	40	5	0	75.7	113	56	16	0	61.1	0.003
**N1**	198	70	116	12	0	58.6	27	96	67	8	48.5	0.044
**N2**	144	40	64	40	0	27.8	34	44	56	10	38.9	0.046
**N3**	83	8	42	28	5	6.0	3	31	42	7	8.4	0.549
**Total**	610	258	262	85	5	49.3	177	227	181	25	44.6	0.096

We reassessed N staging using the 13th edition Japanese anatomic classification [22] due to the relatively poor outcomes of N staging with the 6th edition of UICC TNM staging system [18]. The overall accuracy of N staging using MSCT increased to 67.2%, which was significantly higher than that using the 6th edition UICC N staging and the overall accuracy using EUS (67.2% vs. 44.6% and 49.3%, respectively, P<0.001 for both). The results of N staging using MSCT according to the 13th edition Japanese anatomic classification are summarized in Table 4.

**Table 4:** Accuracy of preoperative N staging by MSCT according to the 13th edition Japanese anatomic classification.

**Pathological stage**	***N***	**MSCT**				**Accuracy %**
		**N0**	**N1**	**N2**	**N3**	
**N0**	185	113	38	33	1	61.1
**N1**	151	22	103	23	3	68.2
**N2**	253	37	32	182	2	71.9
**N3**	21	5	2	2	12	57.1
**Total**	610	177	175	240	18	67.2

The diagnostic sensitivity of MSCT for determination of the presence of metastatic lymph nodes in gastric cancer patients was higher than that of EUS (84.9% vs. 72.2%, P<0.001). However, EUS demonstrated a higher diagnostic specificity compared with MSCT (75.7% vs. 61.1%, P=0.003). Comparisons of EUS and MSCT sensitivity and specificity for metastatic lymph node determinations are presented in Table 5.

**Table 5:** Comparisons of sensitivity and specificity of EUS and MSCT in determining lymph node metastasis.

**Postoperative results**	***N***	**EUS**		**Accuracy %**	**MSCT**		**Accuracy %**	***P* value**
		**N0**	**N+**		**N0**	**N+**		
**N0**	185	140	45	75.7	113	72	61.1	0.003
**N+**	425	118	307	72.2	64	361	84.9	<0.001

### Staging accuracy of distant metastases (M category) using EUS and MSCT

Table 6 summarizes the sensitivity and specificity of EUS and MSCT for the detection of M staging. Overall, the accuracies of EUS and MSCT for M staging were 90.0% and 95.4%, respectively. The accuracy of both techniques was high, but the accuracy of MSCT was statistically higher than that of EUS (P<0.001). EUS was comparably accurate to MSCT for the determination of M0 staging (specificity for M staging) (99.6% vs. 99.8%, P = 1.000), but the accuracy of EUS decreased for the determination of M1 staging (sensitivity for M staging) (10.6% vs. 59.1%, P<0.001). 

**Table 6:** Comparisons of sensitivity and specificity of EUS and MSCT in determining distant metastasis.

**Postoperative results**	***N***	**EUS**		**Accuracy %**	**MSCT**		**Accuracy %**	***P* value**
		**M0**	**M1**		**M0**	**M1**		
**M0**	544	542	2	99.6	543	1	99.8	1.000
**M1**	66	59	7	10.6	27	39	59.1	<0.001
**Total**	610	601	9	90.0	570	40	95.4	<0.001

## Discussion

Our results demonstrated that the overall accuracies of EUS and MSCT for preoperative T staging were 76.7% and 78.2%, respectively. No significant difference between the two modalities was observed. For different levels of staging, we found that the accuracies and advantages of EUS and MSCT differed. We suggest that these differences could help surgeons in decision making when presented with differences between results from EUS and MSCT and that these differences could further elevate the accuracy of preoperative staging. EUS was superior to MSCT for T1 and T2 substaging, but EUS was inferior to MSCT for T3 and T4 substaging. The diagnostic accuracies of EUS and MSCT were poor using the 6th edition UICC N staging. However, the accuracy of MSCT increased notably when N staging was reassessed using the 13th edition Japanese anatomic classification. MSCT exhibited significant advantages in M staging compared with EUS. 

The overall accuracy of MSCT was similar to that of EUS for individual T and N staging in patients with gastric cancer, which is consistent with findings from a previous study [11]. These results suggest that diagnoses of a T1- or T2-staged cancer using EUS and a T-3 or T4-staged cancer using MSCT are preferable when a difference in results occurs. For example, if a tumor were staged as T1 using EUS and T2 using MSCT, then using the EUS result would be recommended. This determination is important because surgeons are often unsure which result is more accurate when a difference in results between EUS and MSCT appears. N staging assessed using MSCT according to the 13th edition Japanese anatomic classification is recommended. This suggestion is meaningful for the following reasons. First, reliable criteria for a definitive diagnosis of metastatic nodes are lacking. Second, quantification of the exact number of metastasized lymph nodes is difficult using radiological methods. Moreover, with regards to therapy management, the anatomical location of the involved lymph nodes is more useful than the exact number.

EUS was superior to MSCT for T1 and T2 staging for the following possible reasons: (1) most early gastric cancers were easily missed because they were small in size with a shallow invasion depth and without obvious enhancement of the mucosa or submucosa; (2) some tumors were difficult to detect because they did not exhibit the typical three-layered structure on CT imaging; (3) some T2 cases were over-staged as T3 because the perigastric adipose space was not as clear on CT imaging, leading to serosal responses being mistaken for invasive cancers; and (4) the key distinction between T1 and T2 was the integrality of the low density zone that corresponded to the submucosa. However, this zone on MSCT scans in some cases was due to edema or fatty deposition, which could have easily distorted the result [23,24]. EUS was advantageous in this respect because we chose ultrasonic probes of different frequencies to increase the accuracy of the assessments. EUS was inferior to MSCT for T3 and T4 staging for the following potential reasons: (1) the thickened gastric wall and/or the protuberant lesions were difficult to detect using the ultrasonic probe, (2) necrotic tissue and fibrosis on the surface of the ulcers decreased the echoes, (3) the gastric wall was difficult to examine because of tumor narrowing, and (4) stomach retention affected the examination. The MSCT was less affected by tumor size, distance, necrotic tissues, and stomach retention. The MSCT incorrectly staged 49 out of 466 T3 category cases. Forty of these cases were under-staged, primarily because MSCT imaging poorly differentiated between early and small tumor invasion in the serosal layer, which is the main differentiating factor between T2 and T3 tumors. The remaining 9 cases were over-staged. Six of these cases were diagnosed with an invasion of the pancreas, and 3 cases were diagnosed with invasions of the right liver lobes. All 9 cases exhibited confirmed inflammatory adhesion instead of tumor invasion on postoperative histopathological examinations. 

The overall accuracies of EUS and MSCT were low, and the clinical significance was limited when N staging was assessed according to the 6th edition UICC classification [18]. Interestingly, the accuracy of EUS and MSCT exhibited a downward trend as the tumor N stage increased. This decrease in accuracy could have been due to the increased difficulty in the exact quantification of metastatic lymph nodes as more lymph nodes became involved as well as the fusion of metastatic lymph nodes. We reassessed the MSCT N stages using the 13th edition Japanese anatomic classification [22], which increased the overall accuracy. Therefore, lymph node metastases should be assessed using MSCT according to the 13th edition Japanese anatomic classification [22].

Our N staging results also demonstrated that MSCT exhibited higher sensitivity than EUS for the estimation of lymph node involvement because EUS was limited by the detection distance. This technique could not evaluate lymph nodes that were distant from the stomach. EUS exhibited greater specificity compared with MSCT. EUS assessments were based on lymph node size, border, shape, hypoechoic pattern and the inner structure, such as the hilum, cortex and medulla. MSCT consistently misdiagnosed metastasized lymph nodes that were very small in size (i.e., smaller in diameter than the scanning layer thickness). Our results revealed that the use of both modalities increased the diagnostic accuracy of lymph node metastases. Specifically, EUS diagnosis is recommended when the EUS is N+ and the MSCT is N0 because the EUS exhibited higher specificity. In contrast, a different recommendation is suggested when the EUS diagnosis is N0 and the MSCT diagnosis is N+. Use of the MSCT result is recommended when the metastatic lymph nodes are located beyond the detection distance of the EUS (5-7 cm) to avoid missed diagnoses. Use of the EUS result is recommended when the metastatic lymph nodes are located within the detection distance of the EUS, which favors a reduction in the number of false-positive results.

We acknowledge that there are limitations to this study. The main purpose of this study was to evaluate and compare the accuracy of preoperative staging using EUS and MSCT as confirmed on post-operative pathological staging. Thus, only patients who were appropriate for open abdominal cancer resection surgery were included, while patients suitable for EMR or ESD or patients with widespread metastatic disease who had not yet undergone surgical resection were excluded. This study was conducted between October 2006 to March 2011, and during that period, the 7th edition of the UICC system (2011) and the most recent Japanese system (2010) had not yet been published. Although the UICC's 6th TNM staging system (2002) and the 13th edition Japanese system (1998) for gastric cancer are no longer used clinically, it is reasonable to use these two systems for preoperative staging of patients, as there is no standard preoperative staging system for gastric cancer. As a result, we used the staging systems released in 2002 and 1998. The main purpose of our study was to compare the accuracy and advantages of EUS and MSCT in different staging of GC; thus, we did not address the impact of obesity and histology on staging modality accuracy. We recommend further investigation of these factors in future studies.

In conclusion, the overall accuracies of EUS and MSCT for the preoperative T staging of gastric cancer were comparable in this study. EUS was clearly superior to MSCT for T1 and T2 staging, but MSCT performed better than EUS for T3 and T4 staging. A global view of the MSCT and EUS results is required to obtain an accurate assessment of metastasized lymph nodes, as both modalities exhibited advantages in either sensitivity or specificity. We suggest that both EUS and MSCT be performed as part of the preoperative evaluation for gastric cancer patients. 
